# Human *U90926* orthologous long non-coding RNA as a novel biomarker for visual prognosis in herpes simplex virus type-1 induced acute retinal necrosis

**DOI:** 10.1038/s41598-021-91340-x

**Published:** 2021-06-09

**Authors:** Shintaro Shirahama, Kenzui Taniue, Shuhei Mitsutomi, Rie Tanaka, Toshikatsu Kaburaki, Tomohito Sato, Masaru Takeuchi, Hidetoshi Kawashima, Yoshihiro Urade, Makoto Aihara, Nobuyoshi Akimitsu

**Affiliations:** 1 Department of Ophthalmology, Graduate School of Medicine, The University of Tokyo, Tokyo, Japan; 2 Isotope Science Center, The University of Tokyo, Tokyo, Japan; 3 Department of Ophthalmology, Jichi Medical University Saitama Medical Centre, Saitama, Japan; 4 Department of Ophthalmology, National Defense Medical College, Saitama, Japan; 5 Department of Ophthalmology, Jichi Medical University, Tochigi, Japan; 6 Daiichi University of Pharmacy, Fukuoka, Japan

**Keywords:** Uveal diseases, Prognostic markers

## Abstract

Acute retinal necrosis (ARN) is a form of infectious uveitis caused by alpha herpesviruses, including herpes simplex virus type 1 (HSV-1). We previously found that the long non-coding RNA (lncRNA) *U90926* is upregulated in murine retinal photoreceptor cells following HSV-1 infection, leading to host cell death. However, to date, an orthologous transcript has not been identified in humans. We investigated *U90926* orthologous transcript in humans and examined its utility as a prognostic marker for visual acuity in patients with ARN. We identified two human orthologous transcripts (1955 and 592 bases) of lncRNA *U90926*. The amount of the longer human *U90926* transcript was approximately 30- and 40-fold higher in the vitreous fluid of patients with ARN than in those with sarcoidosis and intraocular lymphoma, respectively. Furthermore, the expression of the longer human *U90926* transcript in the vitreous fluid was highly correlated with the final best-corrected logarithm of the minimum angle of resolution visual acuity in patients with ARN (r = 0.7671, *p* = 0.0079). This suggests higher expression of the longer human *U90926* transcript in the vitreous fluid results in worse visual prognosis; therefore, expression of the longer human *U90926* transcript is a potential negative prognostic marker for visual acuity in patients with ARN.

## Introduction

Acute retinal necrosis (ARN) is one of the leading causes of blindness in developed countries. It is a type of infectious retinitis caused by members of the alpha herpesvirus subfamily^1^. In a previous study, the main causative viruses of ARN were varicella zoster virus (VZV) (76.1%) and herpes simplex virus (HSV) (15.2%), and ARN caused by HSV was about one-fifth of all cases of ARN caused by VZV^2^. ARN is chiefly diagnosed through viral DNA detection in the intraocular fluid using a high-sensitivity polymerase chain reaction (PCR) assay^1,3^. Although the accurate prediction of visual prognosis is critical to selecting an optimised treatment, biomarkers to predict visual outcomes in patients with ARN are not known. Therefore, it is critical to identify objectively measurable biomarkers that can predict visual prognosis.

Long non-coding RNAs (lncRNAs) are a group of functional RNAs that have diverse biological functions and pathogenic roles, such as immune response^4,5^, pathogenic infection^6,7^, and cancer development^8–11^. In ARN, the neuronal cells of the retina, such as the retinal photoreceptor cells, have been shown to be the primary infected cells^12^. We have previously shown the upregulation of lncRNA *U90926* in murine retinal photoreceptor cells post herpes simplex virus type 1 (HSV-1) infection; this upregulation plays a crucial role in HSV-1 proliferation^13^. However, an orthologous *U90926* transcript has not been identified in humans to date. Therefore, in this study, we investigated the presence of *U90926* orthologous transcripts in humans and examined whether it could be a prognostic marker for visual acuity in patients with ARN caused by HSV-1. Here, we identified two human *U90926* orthologous transcripts and found that the expression of the longer transcript was dramatically upregulated in the vitreous fluid from patients with ARN caused by HSV-1. Furthermore, there was high correlation between the expression of the longer human *U90926* orthologous transcript in the vitreous fluid and the final best-corrected logarithm of the minimum angle of resolution (logMAR) visual acuity. Our results demonstrate that the expression of the longer human *U90926* orthologous transcript could be a potential biomarker for the prognosis of visual acuity in patients with ARN caused by HSV-1.

## Results

### Identification of human *U90926* orthologous transcript

**Figure 1:**
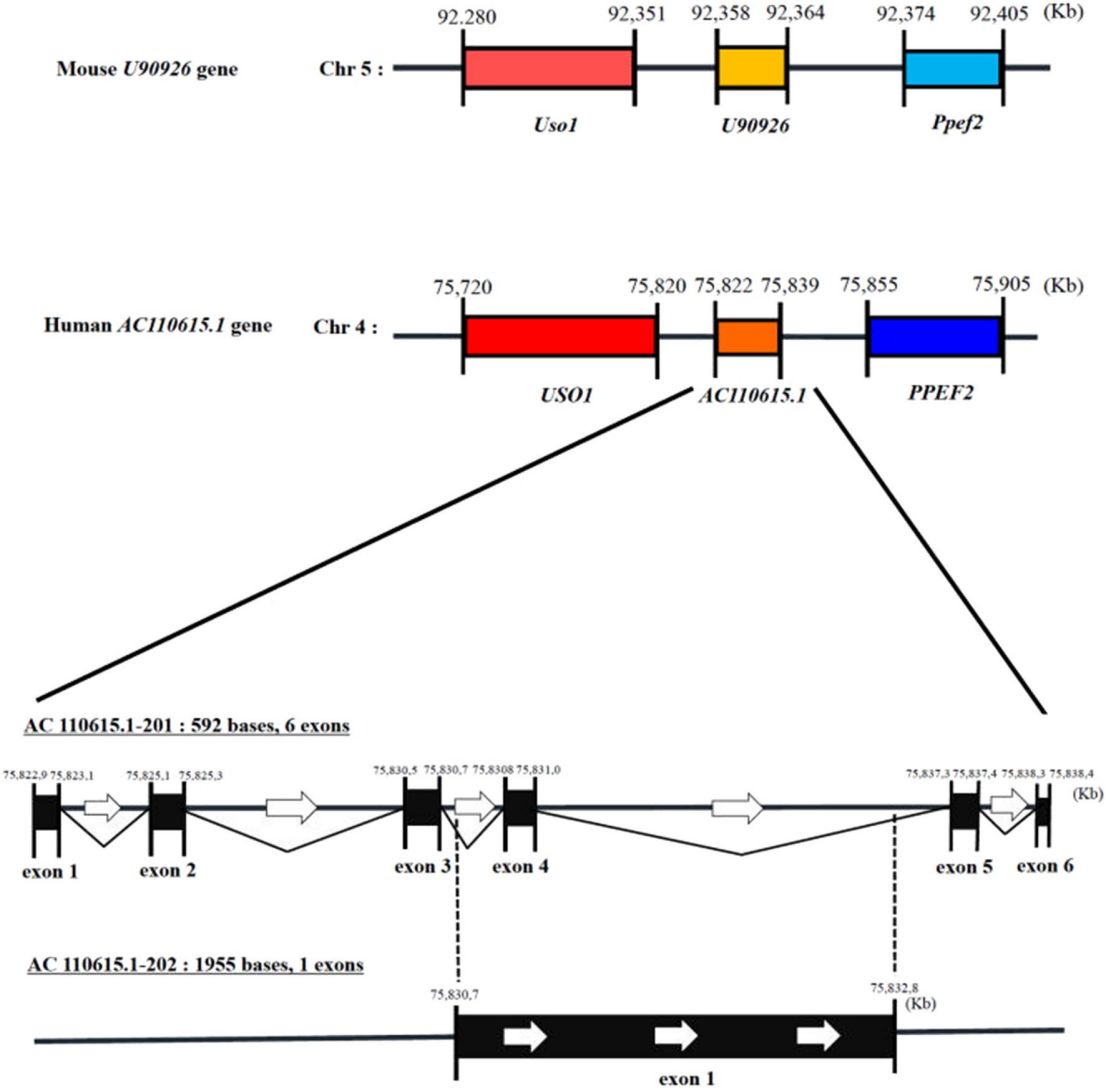
Human *AC110615.1* transcribed from the region between *USO1* and *PPEF2* on chromosome 4. Schematic representation of the genomic organisation of the region containing mouse *U90926* (GRCm38) (top) and human *AC110615.1* (GRCh38) (middle), and that of the composition of human *AC110615.1* transcript (GRCh38) containing *AC110615.1-201* and *AC110615.1-202* (bottom).

To identify the human ortholog of *U90926*, we searched the Ensembl genome browser (www.ensembl.org). The mouse *U90926* gene is located between *Uso1* and *Ppef2* on chromosome 5; this gene is evolutionarily conserved between humans and mice (Fig. 1, top). In the human genome, we found that the *AC110615.1* gene was annotated on the region located between *USO1* and *PPEF2* on chromosome 4 (Fig. 1, middle). We also found that *AC110615.1* encodes short and long variants, termed *AC110615.1-201* (592 bases, 6 exons) and *AC110615.1-202* (1,955 bases, 1 exon), respectively (Fig. 1, bottom). Pairwise sequence alignment revealed a sequence similarity of 45.3% between the mouse *U90926* transcript and *AC110615.1-201* (Supplementary Fig. 1A), and 38.5% between the mouse *U90926* transcript and *AC110615.1-202* (Supplementary Fig. 1B). We named *AC110615.1-201* and *AC110615.1-202* as the short and long human *U90926* transcripts, respectively.

### Upregulation of the long human *U90926* transcript in the vitreous fluid from patients with ARN caused by HSV-1

**Figure 2:**
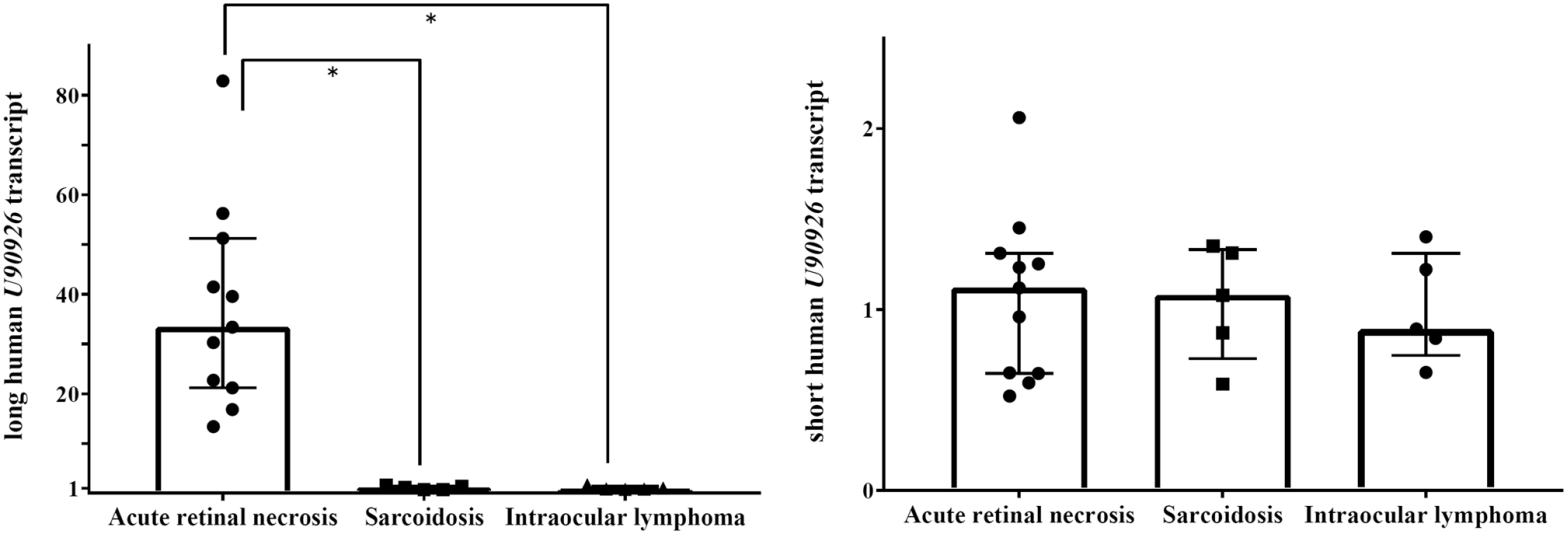
Expression levels of the long and short human *U90926* transcripts in the vitreous fluid from patients with acute retinal necrosis, sarcoidosis, and intraocular lymphoma. A reverse transcription-quantitative real-time polymerase chain reaction analysis of the long (upper) and short (lower) human *U90926* transcripts in the vitreous fluid of patients with acute retinal necrosis (n = 11), sarcoidosis (n = 5), and intraocular lymphoma (n = 5). Results are expressed as mean ± standard deviation. *p < 0.05.

Uveitis is an inflammatory condition of the intraocular tissues, such as the uvea and retina^14^, and it is divided into the following three main categories based on aetiology: infectious uveitis, non-infectious uveitis, and masquerade syndrome^15^. Within these categories, ARN is classified as infectious uveitis. We examined the expression levels of the long and short human *U90926* transcripts in the vitreous fluid of patients with ARN caused by HSV-1 (a type of infectious uveitis), sarcoidosis (a type of non-infectious uveitis), and intraocular lymphoma (a type of masquerade syndrome). Reverse transcription-quantitative real-time PCR (qRT-PCR) analysis revealed that the expression of the long human *U90926* transcript was markedly higher in the vitreous fluid from patients with ARN caused by HSV-1 than from those with sarcoidosis (approximately 30-fold, *p* = 0.0005) or intraocular lymphoma (approximately 40-fold, *p* = 0.0005) (Fig. 2, left). The expression of the short human *U90926* transcript was almost constant among these three patient groups (Fig. 2, right).

### High correlation between the expression of the long human *U90926* transcript in the vitreous fluid and the final best-corrected logMAR visual acuity in patients with ARN caused by HSV-1

**Figure 3:**
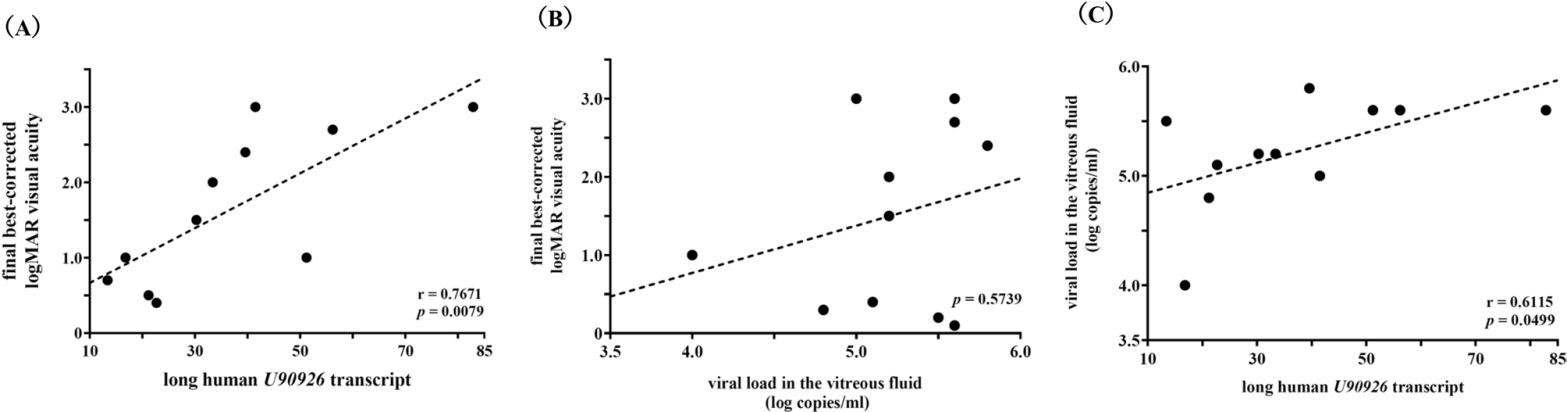
Correlation analyses among the expression of the long human *U90926* transcript, viral loads in the vitreous fluid from patients with acute retinal necrosis caused by herpes simplex virus type 1, and the final best-corrected logarithm of the minimum angle of resolution visual acuity in patients with acute retinal necrosis caused by herpes simplex virus type 1. (**A**) Relationship between the expression of the long human *U90926* transcript in the vitreous fluid and the final best-corrected logarithm of the minimum angle of resolution (logMAR) visual acuity. (**B**) Relationship between viral loads in the vitreous fluid (log copies/mL) and the final best-corrected logMAR visual acuity. (**C**) Relationship between the expression of the long human *U90926* transcript and viral loads (log copies/mL) in the vitreous fluid.

We next examined the relationship between expression of the long human *U90926* transcript and visual prognosis in patients with ARN caused by HSV-1. The expression of the long human *U90926* transcript in the vitreous fluid was highly correlated with final best-corrected logMAR visual acuity in patients with ARN caused by HSV-1 (correlation analysis using Spearman’s rank-correlation coefficient; r = 0.7671, *p* = 0.0079) (Fig. 3A, Supplementary Table 2). In contrast, the viral load in the vitreous fluid was not correlated with the final best-corrected logMAR visual acuity (correlation analysis using Spearman’s rank-correlation coefficient; *p* = 0.5739) (Fig. 3B, Supplementary Table 2). These results indicate that expression of the long human *U90926* transcript in the vitreous fluid is an excellent negative biomarker for the visual outcomes of patients with ARN caused by HSV-1, although the viral load is not useful as a predictor of visual acuity.

### Moderate correlation between the expression of the long human *U90926* transcript and the viral load in the vitreous fluid from patients with ARN caused by HSV-1

The expression of the long human *U90926* transcript in the vitreous fluid of patients with ARN caused by HSV-1 was moderately correlated with the viral load in the vitreous fluid (correlation analysis using Spearman’s rank-correlation coefficient; r = 0.6115, *p* = 0.0499) (Fig. 3C, Supplementary Table 2). This result suggests that expression of the long human *U90926* transcript reflects the viral load in the vitreous fluid.

## Discussion

In this study, we identified long and short human *U90926* transcripts based on high sequence similarities to the mouse *U90926* transcript and found that the long human *U90926* transcript was dramatically upregulated in the vitreous fluid of patients with ARN caused by HSV-1. Furthermore, in patients with ARN caused by HSV-1, we demonstrated that there was a high correlation between the expression of the human long *U90926* transcript in the vitreous fluid and the final best-corrected logMAR visual acuity as well as a moderate correlation between the expression of the long human *U90926* transcript and the viral load in the vitreous fluid.

Retinal photoreceptor cells are divided into two major types: cone and rod cells. In humans, cone cells are responsible for colour vision and high visual acuity. Consequently, the death of cone cells leads to significant vision loss^16^. We previously showed that the mouse *U90926* transcript was dramatically upregulated in 661W cells derived from cone cells post HSV-1 infection^13^. In 661W cells, lncRNA *U90926* acted as a major contributor to host cell death by promoting HSV-1 proliferation^13^. Considering these results, we presumed that upregulated expression of the long human *U90926* transcript in the vitreous fluid reflected an increase in the number of infected cone cells, which in turn induced cone cell death by promoting viral proliferation. Consequently, the expression of the long human *U90926* transcript might reflect the visual prognosis in patients with ARN caused by HSV-1. Although the detailed mechanism underlying this strong correlation between upregulated expression of the long human *U90926* transcript and the final best-corrected logMAR visual acuity remains unclear, our results highlight the usefulness of measuring the expression of the long human *U90926* transcript in assessing the prognosis of visual acuity in patients with ARN caused by HSV-1.

In this study, all patients with ARN caused by HSV-1 were treated with systemic acyclovir therapy before the vitreous fluid was collected. Therefore, we had to consider the possibility that the viral load may have decreased at the time of the vitreous fluid collection due to the acyclovir therapy. However, there was a moderate correlation between the expression of the long human *U90926* transcript and the viral load in the vitreous fluid. If the vitreous fluid prior to systemic acyclovir treatment had been used for analysis, it is possible that there would have been an even stronger correlation between the expression of the long human *U90926* transcript and the viral load in the vitreous fluid.

## Methods

### Sequence alignment

Pairwise sequence alignment was performed using Clustal Omega^17^.

### Patients

This study was approved by the Research Ethics Committee of the Graduate School of Medicine and Faculty of Medicine at the University of Tokyo and National Defense College Hospital [No.10984-(6)]. Written informed consent was obtained from all patients. All procedures were performed in accordance with the principles of the Declaration of Helsinki. This study included a total of 21 eyes of 21 patients (12 males and 9 females; average age = 59.0 ± 18.1 years) who underwent pars plana vitrectomy for the diagnosis and/or treatment of ARN, sarcoidosis, and intraocular lymphoma in the University of Tokyo Hospital and National Defense Medical College Hospital between December 2013 and December 2018. There were 8 patients (11 eyes) diagnosed with ARN caused by HSV-1 (7 males and 4 females; average age = 53.5 ± 16.7 years), 5 patients (5 eyes) with sarcoidosis (2 males and 3 females; average age = 57.0 ± 20.0 years), and 5 patients (5 eyes) with intraocular lymphoma (3 males and 2 females; average age = 73.2 ± 14.0 years). The diagnoses of ARN, sarcoidosis, and intraocular lymphoma were made according to certain diagnostic criteria. A diagnosis of ARN was based on the previously reported criteria for ARN in Japan^3^. For sarcoidosis, we used the diagnostic criteria established by the International Workshop on Ocular Sarcoidosis^18^. An intraocular lymphoma diagnosis was made if at least two of the following four criteria were met: (1) the result of cytological diagnosis based on Papanicolaou’s classification using Papanicolaou-stained cytological preparations was greater than class III^19^; (2) interleukin (IL)-10/IL-6 ratio > 1, or IL-10 > 50 pg/mL in the intraocular fluid^20,21^; (3) strong deviation in the free light chain κ/λ ratio on fluorescence-activated cell sorting analysis^22^; and (4) positive PCR results for immunoglobulin heavy-chain gene rearrangement^23–25^.

### Collection of vitreous fluid and measurement of viral loads in the vitreous fluid

A standard 3-port 23-gauge or 25-gauge vitrectomy was performed, and vitreous fluid was collected aseptically without dilution. The collected vitreous fluid was stored immediately in a pre-sterilised cryotube at − 80 °C. DNA was extracted from each vitreous fluid, and then multiplex quantitative PCR assay for viral DNA (HSV-1, HSV-2, varicella zoster virus, Epstein–Barr virus, cytomegalovirus, human herpesvirus 6, human herpesvirus 7, and human herpesvirus 8) was performed as previously described^26^.

### RNA extraction and quality control

Total RNA from the vitreous fluid was extracted using a NucleoSpin miRNA plasma kit (Macherey–Nagel, Duren, Germany). The assessment of total RNA quality was performed on an Agilent 2100 Bioanalyzer (Agilent Technologies, Santa Clara, CA) using an RNA 6000 Pico Kit (Agilent Technologies). Samples with an RNA integrity number of ≥ 7.0 were used in this study.

### Reverse transcription-quantitative real-time polymerase chain reaction analysis

Total RNA was reverse transcribed into cDNA using Prime Script RT Master Mix (TaKaRa Bio, Shiga, Japan). The cDNA was amplified using both the specific primer sets listed in Supplementary Table 1 and SYBR Premix Ex Taq II (TaKaRa Bio) in accordance with the manufacturer’s instructions. The qPCR was then performed using a Thermal Cycler Dice Real Time System (TaKaRa Bio). *GAPDH* mRNA was used for transcript normalisation. The relative RNA amount of *AC 110615.1* transcript in the vitreous fluid from patients with ARN caused by HSV-1and sarcoidosis was calculated based on that in the vitreous fluid from patients with intraocular lymphoma.

### Measurement of visual acuity

Decimal visual acuity was measured using a Landolt ring, and then converted to logMAR visual acuity using the following formula: logMAR visual acuity = log (1/decimal visual acuity). As previously described^27^, logMAR visual acuity of finger counting, motus manus, and light sense was defined as 2.4, 2.7, and 3.0, respectively. A higher logMAR value indicates lower visual activity.

### Statistical analysis

Values were presented as median with interquartile range. Comparison of continuous variables between the two groups was performed using Mann–Whitney U-test. Correlation between two variables was analysed using Spearman’s rank-correlation coefficient. A *p* value < 0.05 was considered statistically significant. Both Mann–Whitney U-test and correlation analysis were performed using GraphPad Prism version 7 (GraphPad Software, San Diego, CA).

## Supplementary Information

Supplementary Information 1.

Supplementary Information 2.

Supplementary Information 3.

Supplementary Information 4.

Supplementary Information 5.

## Data Availability

The materials and datasets used and analysed during the current study can be obtained from the corresponding author on reasonable request.

## References

[1] Schoenberger SD (2017). Diagnosis and treatment of acute retinal necrosis: A report by the American Academy of Ophthalmology. Ophthalmology.

[2] Sonoda KH (2021). Epidemiology of uveitis in Japan: a 2016 retrospective nationwide survey. Jpn. J. Ophthalmol..

[3] Takase H (2015). Development and validation of new diagnostic criteria for acute retinal necrosis. Jpn. J. Ophthalmol..

[4] Imamura K, Akimitsu N (2014). Long non-coding RNAs involved in immune responses. Front. Immunol..

[5] Imamura K (2014). Long noncoding RNA NEAT1-dependent SFPQ relocation from promoter region to paraspeckle mediates IL8 expression upon immune stimuli. Mol. Cell.

[6] Shirahama S, Miki A, Kaburaki T, Akimitsu N (2020). Long non-coding RNAs involved in pathogenic infection. Front. Genet..

[7] Imamura K (2018). Diminished nuclear RNA decay upon *Salmonella* infection upregulates antibacterial noncoding RNAs. EMBO J..

[8] Tano K (2010). MALAT-1 enhances cell motility of lung adenocarcinoma cells by influencing the expression of motility-related genes. FEBS Lett..

[9] Tano K, Akimitsu N (2012). Long non-coding RNAs in cancer progression. Front. Genet..

[10] Taniue K (2016). Long noncoding RNA *UPAT* promotes colon tumorigenesis by inhibiting degradation of UHRF1. Proc. Natl. Acad. Sci. U S A..

[11] Taniue K (2016). *ASBEL*-TCF3 complex is required for the tumorigenicity of colorectal cancer cells. Proc. Natl. Acad. Sci. U S A..

[12] Pettit TH, Kimura SJ, Uchida Y, Peters H (1965). Herpes simplex uveitis: An experimental study with the fluorescein-labeled antibody technique. Invest. Ophthalmol. Vis. Sci..

[13] Shirahama S (2020). Long noncoding RNA U90926 is crucial for herpes simplex virus type 1 proliferation in murine retinal photoreceptor cells. Sci. Rep..

[14] Kanski JJ, Bowling B (2015). Kanski’s Clinical Ophthalmology: A Systematic Approach.

[15] Deschenes J, Murray PI, Rao NA, Nussenblatt RB, International Unveitis Study Group (2008). International Uveitis Study Group (IUSG): Clinical classification of uveitis. Ocul. Immunol. Inflamm..

[16] Molday RS, Moritz OL (2015). Photoreceptors at a glance. J. Cell Sci..

[17] Sievers F (2011). Fast, scalable generation of high-quality protein multiple sequence alignments using Clustal Omega. Mol. Syst. Biol..

[18] Mochizuki M (2019). Revised criteria of International Workshop on Ocular Sarcoidosis (IWOS) for the diagnosis of ocular sarcoidosis. Br. J. Ophthalmol..

[19] Kaburaki T (2017). Combined intravitreal methotrexate and immunochemotherapy followed by reduced-dose whole-brain radiotherapy for newly diagnosed B-cell primary intraocular lymphoma. Br. J. Haematol..

[20] Cassoux N (2007). IL-10 measurement in aqueous humor for screening patients with suspicion of primary intraocular lymphoma. Invest. Ophthalmol. Vis. Sci..

[21] Whitcup SM (1997). Association of interleukin 10 in the vitreous and cerebrospinal fluid and primary central nervous system lymphoma. Arch. Ophthalmol..

[22] Davis JL, Miller DM, Ruiz P (2005). Diagnostic testing of vitrectomy specimens. Am. J. Ophthalmol..

[23] Baehring JM (2005). Analysis of clonal immunoglobulin heavy chain rearrangements in ocular lymphoma. Cancer.

[24] Sugita S (2009). Diagnosis of intraocular lymphoma by polymerase chain reaction analysis and cytokine profiling of the vitreous fluid. Jpn. J. Ophthalmol..

[25] Ohta K (2007). B cell clonality of primary central nervous system and primary intraocular lymphomas. Jpn. J. Ophthalmol..

[26] Sugita S (2008). Use of multiplex PCR and real-time PCR to detect human herpes virus genome in ocular fluids of patients with uveitis. Br. J. Ophthalmol..

[27] Oshika T (1994). Small Incision Cataract Surgery.

