# Supplementary material for: Human U90926 orthologous long non-coding RNA as a novel biomarker for visual prognosis in herpes simplex virus type-1 induced acute retinal necrosis
Source: Sci Rep. 2021 Jun 9;11:12164. doi: 10.1038/s41598-021-91340-x (PMC8190147; doi:10.1038/s41598-021-91340-x)
Supplement: Supplementary file 1 — Supplementary Information 1. [file 41598_2021_91340_MOESM1_ESM.docx]

**Supplementary Figure 1. Similarity between mouse *U90926* transcript and human *AC110615.1* transcript sequence.**

(A, B) Pairwise sequence alignment between mouse *U90926* transcript and human *AC110615.1-201* (A) and between mouse *U90926* transcript and human *AC110615.1-202* (B). Bases that are homologous between two transcripts are shown in yellow.

**Supplementary Table 1. Primers used in this study.**

**Supplementary Table 2. Clinical information at the time of vitreous fluid collection and raw data of the relative expression of the long human *U90926* transcript, viral loads in vitreous fluid, and final best-corrected logarithm of the minimum angle of resolution visual acuity in each patient with acute retinal necrosis caused by herpes simplex virus type 1.**
